# Assessing the French Interpersonal Reactivity Index (IRI): Psychometric and Qualitative Properties Through the Three French Versions of the IRI Scale

**DOI:** 10.5334/pb.1328

**Published:** 2025-04-02

**Authors:** Giulia Gaggero, Angèle Brunellière, Maria Francesca Gigliotti, Wassila El Mardi, Sylvie Berthoz, Jean-Louis Nandrino, Karyn Doba, Delphine Grynberg

**Affiliations:** 1 Department of Psychology and Cognitive Sciences, University of Trento, Rovereto, Italy; 2 Cognitive and Educational Sciences (CES) Lab, Faculty of Education, Free University of Bolzano-Bozen, Bressanone-Brixen, Italy; 3 CNRS, UMR 9193- SCALab – Sciences Cognitives et Sciences Affectives, Univ. Lille, 59000, Lille, France; 4 Univ. Lille, Inria, CNRS, Centrale Lille, UMR 9189- CRIStAL, F-59000, Lille, France; 5 Laboratoire DysCo – Fonctionnement et Dysfonctionnement Cognitifs : Les âges de la vie, Université Paris 8, Saint-Denis, France; 6 Univ. Bordeaux, INCIA CNRS UMR 5287, 33000 Bordeaux, France; 7 Department of Psychiatry, Institut Mutualiste Montsouris 75014 Paris, France; 8 Institut Universitaire de France, Paris, France

**Keywords:** Interpersonal Reactivity Index, Empathy, French validation, Linguistic adaptation, Confirmatory Factor Analysis, Psychometric properties

## Abstract

The Interpersonal Reactivity Index (IRI) is one of the most used self-report measures of empathy, comprising 4 factors assessing both cognitive and affective empathy. Nowadays, three different French adaptations of this instrument co-exist. This research compares the three French adaptations of the IRI scale using both quantitative and qualitative evaluations. In Study 1, a French-speaking sample (*N* = 339) completed all three French IRI versions at 2-month time intervals in a counterbalanced order. In Study 2, the item wording of the three versions was evaluated by six independent professional translators. Study 1 assessed the items’ distribution, the scale’s factorial structure, the subscales’ internal consistency, and their correlations with alternative measures of empathy (the Empathy Quotient) and other clinically relevant indicators (anxiety, depression). These quantitative analyses highlighted that all three French adaptations can be used for research purposes. They all exhibit acceptable internal consistency, a factorial structure compliant with the 4-factor model originally proposed by Davis, as well as convergent and discriminant validity. However, by combining item quantitative analyses and translators’ judgments, we revealed some problematic items in each version. Taken together, the findings suggest that the French IRI adaptations by Guttman & Laporte (2000) and Braun et al. (2015) should be slightly preferred. To improve the overall quality of each French IRI version, we provide some recommendations about how to adapt problematic items.

## Introduction

Empathy is a broad concept encompassing various phenomena (Batson, 2009). In the psychological literature, it is often conceptualized as a key interpersonal skill, involving the ability to accurately recognize, interpret, and express social communication signals (Stueber, 2008). Thus, empathy represents a central and multifaceted concept bridging developmental, social, and clinical psychology, as well as affective and social neuroscience. Although more than 40 definitions of empathy are currently in use (Cuff et al., 2016), many agree on differentiating empathy into its cognitive and affective components, which characterize the ability to understand another’s feelings and the ability to experience a vicarious emotional response, respectively (Cuff et al., 2016). This distinction has proven relevant, especially in the field of clinical psychology, where deficits in one or more domains of empathy have been associated with several mental disorders. Specific alterations in both cognitive and affective empathy have been found in anorexia nervosa (Doba & Nandrino, 2020; Kerr-Gaffney et al., 2019; Gaggero et al., 2023). Impaired cognitive but preserved affective empathy has been found in both autism spectrum (Harmsen, 2019) and alcohol-use disorders (Maurage et al., 2011). Inversely, there is evidence for impaired affective and preserved cognitive empathy in psychopathy (Campos et al., 2022) and borderline personality disorder (Harari et al., 2010).

In accordance with a two-dimensional conceptualization of empathy (Coke et al., 1978; Feshbach, 1976; Hoffman, 1977), Davis (1980) developed a convenient 28-item self-report scale to measure individual differences in these two components of the construct. The Interpersonal Reactivity Index–IRI (Davis, 1980) comprises four 7-item subscales: Personal Distress (PD), assessing the tendency to experience feelings of distress in tense interpersonal situations; Empathic Concern (EC), assessing the ability to experience other-oriented feelings of sympathy and concern for unfortunate others; Perspective Taking (PT), assessing the ability to understand others’ points of view; and Fantasy (FS), measuring the propensity to become immersed in stories and identify with fictional characters. According to Davis (Davis, 1980; Davis, 1983), PD and EC assess empathy’s *affective* dimension, while PT and FS measure its *cognitive* dimension.

Currently, the IRI is one of the most widely used self-report measures of empathy, adopted by both psychologists and neuroscientists (de Lima & Osório, 2021). Originally developed in American English, the IRI has been translated and validated in many other languages, including Chinese (Siu & Shek, 2005), Dutch (De Corte et al., 2007), Farsi (Golbabaei et al., 2022), French (Braun et al., 2015; Gilet et al., 2013; Guttman & Laporte, 2000), German (Paulus, 2009), Italian (Albiero et al., 2006), Japanese (Himichi et al., 2017), Korean (Kang et al., 2016), Spanish (Ochotorena et al., 2003), and Swedish (Cliffordson, 2002). There exist three versions of the IRI scale adapted for French speakers, each independently developed by different research teams from three different French-speaking countries (in Canada by Guttman & Laporte, 2000; in Switzerland by Gilet et al., 2013; in Belgium by Braun et al., 2015). Crucially, the three versions exhibit substantial variations in item wording as well as variations in the rating scale.

The first French version of the IRI scale (Guttman & Laporte, 2000) was developed by researchers working at Canadian universities and was initially tested on women with borderline personality disorder (n = 27), women with anorexia nervosa (n = 28), and women without a clinical diagnosis (n = 27), as well as their families. With a total sample of 325 French-speaking Canadian adults, the study reported a mean internal consistency ranging from 0.63 to 0.70 for the four IRI subscales, which indicates relatively low reliability. However, no proper validation analyses were conducted (e.g., factorial structure, convergent/divergent validity analyses) since its main focus was only on comparing the empathy scores of the examined clinical populations.

The second version (Gilet et al., 2013) was created by researchers working at French and Swiss universities. Notably, the authors changed the rating scale from a 5-point to a 7-point Likert scale, justifying their choice with the aim of improving scale sensitivity. This version was validated with a sample of 322 French-speaking Swiss adults. Internal consistency ranged from 0.70 to 0.81, resulting in being better than Guttman & Laporte’s version. Authors performed also confirmatory factorial analyses, reported gender and age differences in subscale scores and correlations with the Empathy Quotient questionnaire (EQ; Baron-Cohen & Wheelwright, 2004; Berthoz et al., 2008), another well-established scale used to assess empathic deficits in clinical populations such as those with autism.

The third and most recent French translation of the IRI was provided by researchers working at Belgian universities (Braun et al., 2015). This version’s psychometric properties were tested in two large French-speaking Belgian student samples (N_1_ = 1244, N_2_ = 729). Internal consistency ranged from 0.65 to 0.76 for the four subscales. The authors provided a factorial analysis and proposed a shortened 15-item version of the questionnaire. This shortened version presented good fit indexes when tested for Davis’ 4-factor model, although Cronbach’s alpha values for two scales (EC and PT) were still lower than 0.70. A summary of the diverse psychometric properties of the original English and the three French versions can be found in the Supplementary Material (Table S1).

The existence of three different French translations of the IRI represents a source of confusion for researchers and clinicians aiming to administer the scale to a French-speaking population. To date, studies using French adaptations of the IRI select a version without providing an explanation of the rationale behind their choice or without even acknowledging the existence of multiple French versions. Additionally, since the studies validating the three French IRI versions (Braun et al., 2015; Gilet et al., 2013; Guttman & Laporte, 2000) used non-comparable samples (differing in size, clinical history, age, and professional status), drawing meaningful comparisons between the results of these French IRI scales is challenging. Structural validity (factorial structure) was not tested in all samples, and convergent/divergent validity was either not measured or was assessed using different scales (see Table S1). These disparities make it impossible to compare the versions, preventing researchers from making an informed decision about which adaptation to use and emphasizing the necessity to clarify whether the three IRI versions have different associations with similar or different constructs.

This research aimed to compare the psychometric properties (Study 1) and specific item wording (Study 2) of the three French versions of the IRI scale. Our research contribution lies in 1) the involvement of a unique French-speaking sample, completing all three versions, to address the issue of sample heterogeneity in previous validation studies; 2) a detailed confrontation of the three versions, describing and summarizing all the methodological choices made by their respective original authors; 3) a systematic qualitative and quantitative comparison of the three versions, consisting in the evaluation of psychometric properties and item wording of the three versions; 4) the evaluation of convergent/divergent validity based on correlation with other constructs for all the three versions. With this multimethod approach, we intend to provide guidance for researchers in the selection of an IRI French translation that demonstrates the best linguistic and emotional equivalence with the original English version and that most strongly complies with psychometric standards. Further, we highlight the strengths and weaknesses of each item’s wording and provide valuable recommendations to improve the validity of each version.

## Study 1

Study 1 evaluated the psychometric properties of the three French versions of the IRI within the same French-speaking sample. The procedure we used replicates previous studies comparing different cross-cultural adaptations of the same self-report instrument (Boussat et al., 2021; Perneger et al., 1999). In compliance with scale evaluation best practices (Boateng et al., 2018; Cheung et al., 2024), we assessed internal/structural validity, measurement invariance across gender, and convergent/discriminant validity to evaluate how strong the IRI versions assess similar or different construct than other reference scales.

Convergent validity was assessed by correlating IRI subscales with the EQ (Baron-Cohen & Wheelwright, 2004), a self-report questionnaire designed for application in clinical contexts. The EQ was chosen as two previous studies, each using different French adaptations of the IRI (Berthoz et al., 2008; Gilet et al., 2013), reported different correlation indexes between some IRI subscales (PD, FS) and EQ scores. Such mixed findings suggest that further evidence is needed to better determine the associations between the constructs targeted by the IRI and the EQ and understand the reason behind the divergence of any of the three French versions.

Finally, divergent validity was measured using depression and trait-anxiety self-report questionnaires, based on the hypothesis that cognitive empathy dimensions (PT and FS) would present low or non-significant correlations with depression and anxiety measures, while affective empathy (especially PD) would be positively correlated with them. This hypothesis relies on results from correlation analyses of previous studies with French-speaking non-clinical and clinical populations and meta-analyses conducted across several national samples (Achim et al., 2011; Berthoz et al., 2008; Fernandez et al., 2011; Gaggero et al., 2023; Grynberg et al., 2010; Nair et al., 2024; Yan et al., 2021). Further, it aligns with the theoretical framework of empathy proposed by Batson et al. (1987), who dissociated cognitive components of empathy from personal distress. As personal distress corresponds to an individual’s levels of discomfort and anxiety when witnessing others’ negative experiences, those with a high level of distress—in terms of anxiety and depression symptomatology—might be at risk for reporting higher levels of personal distress. Thus, following Batson et al.’s theory, distress does not represent empathy or concern since the main goal of the distressed person is to reduce this uncomfortable state, sometimes even by avoiding the distressing situation/other person when the cost of avoidance is limited (Batson et al., 1987).

To summarize, anxiety and depression measures were selected because these variables can simultaneously evaluate the divergent validity of the cognitive dimensions and the convergent validity of the affective dimensions of the IRI and, more specifically, PD. Furthermore, we aimed to test whether the association between the affective dimensions differed among the French adaptations. Researchers might prefer a version that is less influenced/confounded by anxiety and depression symptoms. Additionally, because the IRI is frequently used to examine empathy deficits in clinical populations characterized by higher levels of anxiety and/or distress (e.g., schizophrenia, alcohol use disorders, autism spectrum disorder; Achim et al., 2011; Hollocks et al. 2019; Smith & Randall, 2012), the comparison with such measures could be useful.

### Materials and Methods

#### Participants

The analyses included 339 participants (261 women, 71 men, 7 others; *M_age_* = 29.35, *SD_age_* = 13.19, age range = 18–72; 55% university students, 39% workers, 6% other). A total of 374 other participants took part in the experiment but were excluded since they only participated in one or two phases. Retained participants completed all three phases of the experiment and met the pre-registered inclusion criteria (see below). Our sample size was compliant with the minimum size declared in the pre-registered protocol (*N* = 336). The required sample size was computed by adopting a conservative heuristic applied for running factorial analyses (i.e., 12 observations per item). The pre-registered inclusion criteria were a minimum age of 18 years old and a residence in France. Participants also had to be native French speakers or, if not, they had to self-evaluate their level of French language competence as excellent (equivalent to scoring five on a 5-point Likert scale). Two participants who scored below this threshold were excluded. Three additional participants were excluded since they were identified as extreme outliers (>97.5%) with patterns of identical repeated answers using the *longstring* function from the careless R package (Yentes & Wilhelm, 2018). The attrition rate was therefore 52.08%.

Of the final sample included in the analyses, 21.5% (73 of 339 participants) declared having previously received a diagnosis of mental disorder. Although we initially proposed to exclude participants with a diagnosis of a clinical disorder, we decided to retain this group after verifying that in all measures, their responses to the questionnaires exhibited acceptable reliability values (Cronbach’s alphas > 0.70) and that there was no statistical difference in mean scores among the three groups for all IRI subscales (see Tables S2 and S3 of the Supplementary Material).

#### Procedure

For Study 1, participants completed an online survey comprising the three French versions of the IRI (Braun et al., 2015; Gilet et al., 2013; Guttman & Laporte, 2000). The survey link was advertised through the social network of co-authors and among university students. Following a within-subjects design, the study had three phases. In the first phase, participants completed one of the three French versions of the IRI and a selection of other scales assessing anxiety, depression, and empathy (see below for more details). Demographic information was also collected in this phase. In the second and third phases, occurring 2 months apart, participants were asked to complete the other French versions of the IRI. The completion order of the three versions of the IRI was, thus, counterbalanced across participants using a balanced Latin square design, with six possible ordered lists (*example*: Phase 1: Gilet, Phase 2: Braun, Phase 3: Guttman & Laporte), each automatically assigned by Qualtrics. The mean number of participants for each combination was 56.14 (*SD* = 3.53). Data from the three phases were attributed to the same individual using an anonymization code. Informed consent was provided online, and participants received no monetary compensation. A CNIL (Commission Nationale de l’Informatique et des Libertés) declaration was made by the University’s Data Protection Officer and was registered under the number #2022-094. The study was carried out in accordance with the principles stated in the Declaration of Helsinki. Before data collection, the protocol of this study was pre-registered in the Open Science Framework (https://osf.io/9puvx).

#### Measures

Participants completed the three existing 28-item French versions of the Interpersonal Reactivity Index (IRI; Davis, 1983; French versions: Braun et al. 2015; Gilet et al., 2013, and Guttman & Laporte, 2000). For all three versions, we applied the same 5-point Likert scale ranging from 1 (*“ne me décrit pas vraiment”/“Does not describe me well”*) to 5 (*“me décrit très bien”/“Describes me very well”*) to prevent any effects due to the use of different scales and to determine the potential effect of item wording. The 5-point Likert scale was chosen in accordance with the original American English version.

The other clinical scales administered to the participants were:

**Spielberger’s Trait Anxiety Inventory** (STAI-T; Spielberger et al., 1983; French version: Bruchon-Schweitzer & Paulhan, 1993). This self-report questionnaire includes 20 anxiety-related sentences scored on a Likert scale ranging from 1 (“*Almost Never*”) to 4 (“*Almost Always*”). The final total score ranges from 20 to 80 and indicates the level of the participant’s general anxiety (trait anxiety). In our sample, reliability values were as follows: Cronbach’s α = 0.92, McDonald’s ω = 0.94.

**13-item Beck Depression Inventory** (BDI-13; Beck & Beamesdefer, 1974; French version: Collet & Cottraux, 1986). This scale includes 13 statements assessing how an individual felt over the past 7 days. Each statement is scored on a Likert scale ranging from 0 (“*No symptoms*”) to 3 (“*More severe symptoms*”). A total score ranging from 0 to 39 provides the self-reported level of depression. In our sample, reliability values were as follows: Cronbach’s α = 0.88, McDonald’s ω = 0.90.

**Empathy Quotient Questionnaire** (EQ; Baron-Cohen & Wheelwright, 2004; French version: Berthoz et al., 2008). The EQ is a 60-item questionnaire, with 40 items assessing empathy and 20 filler items. Each item is scored on a 4-point Likert scale ranging from 1 (*“Strongly Agree”*) to 4 (*“Strongly Disagree”*). A total score (EQ Total) ranging from 0 to 80 is computed. In line with previous studies (Lawrence et al., 2004), we also computed three additional scores indexing three EQ factors, namely Cognitive Empathy (EQ-CE), Emotional Reactivity (EQ-ER), and Social Skills (EQ-SS). In our sample, reliability values were as follows: EQ Total: Cronbach’s α = 0.86, McDonald’s ω = 0.87; EQ-CE: Cronbach’s α = 0.89, McDonald’s ω = 0.91; EC-ER: Cronbach’s α = 0.83, McDonald’s ω = 0.80; EQ-SS: Cronbach’s α = 0.36, McDonald’s ω = 0.69.

#### Data analysis

Data analysis was performed using the RStudio software (version 2023.06.2 + 561). Based on previous studies comparing cross-cultural adaptations (Boussat et al., 2021; Perneger et al., 1999), we computed several psychometric indicators in each IRI version, which are detailed below. For all analyses, the significance level was set at 0.05.

##### Descriptive Statistics and Analysis of Variance of the IRI Scores

Mean, standard deviations, floor and ceiling effect (% of individuals who obtain either minimum or maximum scores) for the four IRI subscales (EC, PT, FS, PD) were computed for each version. Non-parametric analyses of variance (Friedman tests) and subsequent post-hoc analyses using Wilcoxon tests and the Bonferroni correction method were applied to compare subscale means (PD, EC, PT, FS) across the three versions. Skewness and kurtosis of each item were also detected to individuate problematic items (i.e., distribution) in each version.

##### Internal Consistency

For each version, Cronbach’s alphas and McDonald’s omegas of the four IRI subscales (EC, PT, FS, PD) were computed. Internal consistency indexes relating to the same subscale were then compared across the three versions using the “cocron” R package (Diedenhofen & Musch, 2016).

##### Factorial Structure

Confirmatory factorial analysis (CFA) was performed using the R package “lavaan” on the score obtained for each item. Similar to a recent meta-analytic factorial study on the IRI (Raimondi et al., 2023), factors were estimated using a “robust” estimator (the weighted least square mean and variance adjusted; WLSMV). CFA was run to reproduce the original 4-factor model proposed by Davis (1983). Model-fit indexes (χ*^2^*, CFI, TLI, RMSEA, SRMR) of the CFA were computed and compared across the three French versions. In line with previous studies’ recommendations (Brown, 2015; Hu & Bentler, 1995), model fit was considered acceptable when indexes were at the following thresholds: RMSEA ≤ 0.08, SRMR ≤ 0.08, CFI ≥ 0.90, GFI and TLI ≥ 0.95. For each version, we also detected the number of items that showed unacceptably low loadings (< .40).

##### Convergent and Divergent Validity

To assess convergent and divergent validity we computed Spearman’s correlation coefficients between the scores for each IRI subscale and for the other clinical scales (anxiety – STAI-T total score, depression – BDI total score, and empathy scores – EQ total and subscale scores). The magnitude of the correlation coefficients was evaluated based on Schober et al.’s (2018) guidelines, indicating weak (0.10–0.39), moderate (0.40–0.69), strong (0.70–0.89), or very large (≥0.90).

##### Gender Differences

Only the female and male groups were considered, as the “other” group displayed a too small sample size (7 participants out of 339). Measurement invariance (configural, metric, scalar) across the two groups was tested using the “lavaan” and “semTools” packages. To compare the different models (configural, metric, scalar), the following goodness of fit indices were considered: Δχ2, ΔCFI (robust), ΔTLI (robust). Gender differences in the scores of each IRI subscale across the three French versions were compared using a non-parametric permutation *t*-test implemented in the “RVAideMemoire” R package (Hervé, 2022).

### Results

#### Descriptive Statistics and Analysis of Variance of the IRI Scores

Table 1 reports descriptive statistics and the analysis of variance of the IRI scores in the three French versions. Friedman tests revealed significant differences in the mean PD, EC, and PT scores among the three French versions. However, post-hoc comparisons revealed that only the mean score for the Personal Distress (PD) subscale was significantly higher in Gutman and Laporte (2000) than in Gilet et al. (2013). Other comparisons were not statistically significant. The percentage of participants showing floor or ceiling effects in the four subscales was negligible for all three versions (<5%). The analysis of items’ skewness and kurtosis also revealed that most items approached a normal distribution with some exceptions, especially for items #12 and #18 (both reversed items), which showed kurtosis or skewness > |1| in all three versions (see Table 2 for details). This might indicate that these items, independently from their translations, are prone to elicit biased answers because they evoke morally (un)desirable behaviors (e.g., item #18 “*When I see someone being treated unfairly, I sometimes don’t feel very much pity for them”*) or culturally (un)desirable behaviors (item #12 *“Becoming extremely involved in a good book or movie is somewhat rare for me”*). Overall, when counting the number of items with high skewness and kurtosis, Gilet’s version presented the highest number of items (three), while Guttman & Laporte’s version presented no items with both high skewness and kurtosis.

**Table 1:** Analysis of Variance of IRI scores in the Three French Versions.


SUB-SCALE	MEAN (SD)			FRIEDMAN TEST	POST-HOC	CEILING EFFECT (%)			FLOOR EFFECT (%)		
		
	G&L	GILET	BRAUN			G&L	GILET	BRAUN	G&L	GILET	BRAUN

PD	14.40 (4.99)	12.90 (5.36)	13.50 (4.94)	χ*²* = 39.02, *p* < .001	G&L > Gilet**	.003	.003	.003	.006	.009	.009

EC	20.00 (4.63)	20.50 (4.39)	19.80 (4.54)	χ*²* = 14.45, *p* < .001	*ns*	.024	.021	.021	.006	.003	.003

PT	18.40 (4.83)	18.90 (4.72)	19.00 (4.60)	χ*²* = 13.90, *p* < .01	*ns*	.015	.015	.027	.003	.003	.003

FS	19.40 (5.63)	19.20 (5.43)	19.30 (5.52)	χ*²* = 4.31, *p* = .12	*ns*	.044	.029	.035	.003	.003	.006


*Note*. G&L = IRI version by Guttman & Laporte, 2000; Gilet = IRI version by Gilet et al., 2013; Braun = IRI version by Braun et al., 2015. EC = Empathic Concern subscale; PD = Personal Distress subscale; PT = Perspective Taking subscale; FS = Fantasy subscale. Friedman test (a non-parametric version of one-way RM ANOVA) was applied to compare the mean distributions of the three versions. Wilcoxon Signed-Rank test was applied for post-hoc comparisons with Bonferroni correction method.

**Table 2:** Items’ Responses Skewness and Kurtosis for the three French Versions of the Interpersonal Reactivity Index.


ITEM	G&L		GILET		BRAUN	
		
	SKEWNESS	KURTOSIS	SKEWNESS	KURTOSIS	SKEWNESS	KURTOSIS

IRI 1	–0.84	–0.21	–0.70	–0.52	–0.95	0.19

IRI 2	–0.53	–0.24	–0.43	–0.41	–0.24	–0.53

IRI 3	–0.65	–0.46	–0.84	–0.10	–0.82	–0.15

IRI 4	–0.48	–0.70	–0.33	–1.00	–0.67	–0.46

IRI 5	–0.58	–0.46	–0.80	0.04	–0.65	–0.36

IRI 6	–0.11	–0.87	–0.25	–0.89	–0.44	–0.54

IRI 7	–0.91	0.38	–0.76	–0.14	–0.47	–0.63

IRI 8	–0.72	0.11	–0.93	**1.20**	–0.82	**1.12**

IRI 9	–0.67	0.42	–0.90	0.98	–0.87	**1.13**

IRI 10	–0.53	–0.71	–0.61	–0.52	–0.67	–0.39

IRI 11	–0.76	0.58	–0.92	0.71	–0.97	**1.12**

IRI 12	**–1.22**	0.70	**–1.56**	**2.17**	**–1.20**	0.70

IRI 13	–0.62	–0.02	0.19	–0.64	0.28	–0.58

IRI 14	–0.94	0.35	**–1.28**	**1.46**	–0.68	–0.04

IRI 15	–0.32	–0.67	–0.52	–0.51	–0.5	–0.50

IRI 16	–0.39	**–1.15**	–0.03	**–1.24**	–0.44	–0.97

IRI 17	–0.21	**–1.06**	–0.38	–0.93	–0.38	–0.93

IRI 18	**–1.08**	0.55	**–1.90**	**3.40**	**–1.53**	**2.34**

IRI 19	0.52	0.12	0.35	–0.42	0.49	–0.06

IRI 20	–0.44	–0.23	–0.74	0.15	–0.67	0.11

IRI 21	–0.53	–0.21	–0.62	0.09	–0.56	–0.31

IRI 22	–0.77	–0.09	–0.80	0.33	–0.78	0.13

IRI 23	–0.74	–0.24	–0.87	0.25	–0.81	–0.10

IRI 24	0.64	–0.12	0.45	–0.45	0.52	–0.31

IRI 25	–0.02	**–1.08**	0.03	–0.91	–0.21	–0.76

IRI 26	–0.88	–0.06	–0.75	–0.15	–0.77	–0.45

IRI 27	0.42	–0.47	**1.01**	0.83	0.79	0.59

IRI 28	–0.35	–0.52	–0.44	–0.34	–0.61	–0.07

N items >|1|	2	3	4	5	2	4

Abs. Mean	0.53	0.40	0.41	0.97	0.52	0.83

PD	0.18	–0.29	–0.05	–0.50	–0.05	–0.31

EC	–0.58	–0.18	–0.57	–0.07	–0.72	0.65

PT	–0.31	–0.28	–0.41	–0.02	–0.32	–0.08

FS	–0.54	–0.15	–0.63	0.04	–0.48	–0.42


*Note*. G&L = IRI version by Guttman & Laporte, 2000; Gilet = IRI version by Gilet et al., 2013; Braun = IRI version by Braun et al., 2015. EC = Empathic Concern subscale; PD = Personal Distress subscale; PT = Perspective Taking subscale; FS = Fantasy subscale; Abs. Mean = Absolute Mean; Values > 1.00 or < –1.00 are highlighted in bold.

#### Internal Consistency

Table 3 displays Cronbach’s alphas and McDonald’s omegas for each subscale of the three French versions of the IRI. All values were above >0.70, indicating good internal consistency for all. No differences in Cronbach’s alphas were found when comparing the three versions (PD: χ^2^(2) = 3.15, *p* = 0.207; EC: χ^2^(2) = 2.51, *p* = 0.285; PT: χ^2^(2) = 0.18, *p* = 0.913; F: χ^2^(2) = 1.06, *p* = 0.589). Item-total and inter-item correlations for each version are also displayed in Tables S4 and S5 of the Supplementary Material.

**Table 3:** Internal Consistency (Cronbach’s Alpha and McDonald’s Omega) of the Three French Versions.


SUBSCALES	G&L	GILET	BRAUN

PD	α = .79, ω = .89	α = .83, ω = .89	α = .80, ω = .86

EC	α = .79, ω = .84	α = .75, ω = .82	α = .79, ω = .83

PT	α = .79, ω = .85	α = .79, ω = .86	α = .78, ω = .85

FS	α = .83, ω = .88	α = .81, ω = .87	α = .81, ω = .87


*Note*. G&L = IRI version by Guttman & Laporte, 2000; Gilet = IRI version by Gilet et al., 2013; Braun = IRI version by Braun et al., 2015. EC = Empathic Concern subscale; PD = Personal Distress subscales; PT = Perspective Taking subscale; FS = Fantasy subscale.

#### Factorial Structure

Table 4 presents the fit indexes yielded by the CFA with the WLSMV estimator when reproducing the original 4-factor model proposed by Davis (1983). All versions showed comparable fit indexes. The majority of indexes fully met the recommended criteria for a good fit, with the only exceptions being GFI and TLI, which were slightly lower than the recommended criteria but overall acceptable. Table 5 shows the items’ factor loadings. In Gilet’s version, two items (items #18 and #1) resulted in a factor loading below 0.40. In Braun’s version, one item (item #1) presented a factor loading below 0.40. In Guttman & Laporte’s version, two items (items #13 and #4) had a factor loading lower than 0.40. Notably, item #1 (“*I daydream and fantasize, with some regularity, about things that might happen to me*”) had low factor loadings in all three French versions, suggesting that the item does not fit well within the FS factor. This could be due to wording problems that are not limited to a particular version, such as the non-specification of the adaptive/maladaptive type of daydreams and fantasies.

**Table 4:** Fit Indexes yielded by the 4-Factor Confirmatory Factor Analysis of the Three French Versions.


FIT INDEXES	G&L	GILET	BRAUN	RECOMMENDED CRITERIA OF GOOD FIT

Chi-squared (χ^2^)	643.20***	596.30***	589.87***	significant p-value

Root Mean Square Error of Approximation (RMSEA)	0.05	0.05	0.05	<0.08

Comparative Fit Index (CFI)	0.94	0.95	0.94	≥0.90

Tucker-Lewis Index (TLI)	0.93	0.94	0.94	≥0.95

Standardized Root Mean Residual (SRMR)	0.07	0.07	0.07	<0.08

Goodness of Fit Index (GFI)	0.94	0.94	0.94	≥0.95


*Note*. G&L = IRI version by Guttman & Laporte, 2000; Gilet = IRI version by Gilet et al., 2013; Braun = IRI version by Braun et al., 2015. *** = *p* <. 001.

**Table 5:** Confirmatory Factorial Analysis (CFA). Factor Loadings compared for the three French Versions of the IRI.


ITEM	G&L				GILET				BRAUN			
		
	PD	EC	PT	FS	PD	EC	PT	FS	PD	EC	PT	FS

IRI 6	.76				.73				.63			

IRI 10	.59				.55				.65			

IRI 13	**.22**				.55				.51			

IRI 17	.54				.53				.55			

IRI 19	.61				.67				.55			

IRI 24	.79				.86				.72			

IRI 27	.66				.63				.61			

IRI 2		.70				.72				.57		

IRI 4		**.37**				.58				.65		

IRI 9		.40				.47				.40		

IRI 14		.73				.64				.73		

IRI 18		.66				**.36**				.44		

IRI 20		.69				.46				.66		

IRI 22		.58				.65				.68		

IRI 3			.55				.59				.65	

IRI 15			.50				.58				.49	

IRI 8			.61				.58				.55	

IRI 11			.74				.63				.73	

IRI 21			.58				.51				.40	

IRI 25			.62				.62				.64	

IRI 28			.61				.66				.64	

IRI 1				.42				**.38**				**.38**

IRI 5				.69				.74				.73

IRI 7				.59				.58				.51

IRI 12				.58				.52				.44

IRI 16				.70				.67				.72

IRI 23				.84				.79				.80

IRI 26				.64				.69				.73


*Note*. G&L = IRI version by Guttman & Laporte, 2000; Gilet = IRI version by Gilet et al., 2013; Braun = IRI version by Braun et al., 2015. EC = Empathic Concern subscale; PD = Personal Distress subscales; PT = Perspective Taking subscale; FS = Fantasy subscale. Bold values indicate loadings < .40.

#### Convergent and Divergent Validity

Table 6 presents the correlations between the IRI and the other psychological scales. Results were comparable across the three versions. Concerning convergent validity, the EQ total scores showed a moderate correlation with EC (0.55–0.57) and PT (0.41–0.48), and a weak correlation with FS (0.20–0.22) for all three versions. Conversely, EQ total scores were not significantly correlated with PD. Concerning divergent validity, anxiety scores (STAI) were moderately correlated with PD scores (0.46–0.49), weakly correlated with FS scores (0.20–0.22), and not significantly correlated with EC or PT. Depression (BDI) scores showed only a weak correlation with PD (0.29–0.34).

**Table 6:** Spearman Correlation Coefficients between the IRI Subscales and Other Scales.


SCALES	EC			PD			PT			FS		
			
	G&L	GILET	BRAUN	G&L	GILET	BRAUN	G&L	GILET	BRAUN	G&L	GILET	BRAUN

STAI Trait	0.06	0.08	0.12	0.50***	0.46***	0.52***	–0.14	–0.08	–0.08	0.22**	0.21**	0.20*

BDI	–0.06	0.01	0.02	0.30***	0.29***	0.34***	–0.06	0.00	–0.03	0.15	0.18	0.16

EQ Total	0.57***	0.57***	0.55***	–0.03	–0.08	–0.02	0.41***	0.47***	0.48***	0.20*	0.22**	0.20*

EQ Cognitive Empathy	0.32***	0.35***	0.34***	–0.14	–0.17	–0.10	0.30***	0.33***	0.31***	0.18	0.17	0.17

EQ Emotional Reactivity	0.66***	0.64***	0.64***	0.10	0.07	0.11	0.35***	0.43***	0.47***	0.27***	0.29***	0.27***

EQ Social Skills	0.17	0.11	0.08	–0.26***	–0.27***	–0.31***	0.18	0.18	0.18	–0.06	–0.06	–0.06


*Note*. EC = Empathic Concern subscale; PD = Personal Distress subscale; PT = Perspective Taking subscale; FS = Fantasy subscale. G&L = IRI version by Guttman & Laporte, 2000; Gilet = IRI version by Gilet et al., 2013; Braun = IRI version by Braun et al., 2015. STAI-T = Spielberger’s Trait Anxiety Inventory; BDI = Beck Depression Inventory; EQ = Empathy Quotient. * = p <. 05; ** = *p* <. 01; *** = *p* <. 001.

#### Gender Differences

Concerning measurement invariance across genders (*N*_female_ = 261, *N*_male_ = 71), all three versions exhibited similar psychometric properties when moving toward models with more constraints. Indeed, Table S6 of the Supplementary Material illustrates that (a) goodness of fit indices (χ2, CFI robust, TLI robust, RMSEA) in configural invariance models were at acceptable levels (CFI robust and TLI robust ranged between 0.89 and 0.92), (b) the Δχ2 statistics varied across versions and indicated a lack of scalar invariance in Braun and Gilet versions. However, Δχ2 is usually significant with more complex models and large sample sizes; on the contrary, (c) all ΔCFI and ΔTLI were lower than 0.01, suggesting both metric and scalar invariance. These results partially legitimize the between-group comparison by gender. Results from non-parametric permutation *t*-tests revealed significantly higher IRI EC scores in women (*M*_G&L_ = 20.56, *SD*_G&L_ = 4.42; *M*_Gilet_ = 21.01, *SD*_Gilet_ = 4.24; *M*_Braun_ = 20.36, *SD*_Braun_ = 4.40) than men (*M*_G&L_ = 17.79; *SD*_G&L_ = 4.80; *M*_Gilet_ = 18.30, *SD*_Gilet_ = 4.38; *M*_Braun_ = 17.69, *SD*_Braun_ = 4.55) across all the three French versions (*p* = .002, *p_adj_* = .007). Gender differences in the other IRI subscales were not significant after correcting for multiple comparisons. All results are presented in Table S7 of the Supplementary Material.

## Study 2

In Study 2, we obtained valuable information about the quality of item wording from the evaluations of six professional translators. Qualitative data on item wording quality are usually considered at the stage of scale development in order to generate and refine items. Consulting with both experts in the field and the target population is part of the best practices adopted to guarantee the content validity of a measure (Boateng et al., 2018). When validating a scale translation, the content validity is already taken for granted. Nevertheless, linguistic adaptations should guarantee that the meaning and intensity of the adapted translation are equivalent to those of the original. This is usually guaranteed by the back-translation process. However, the existence of three different French translations of the same instrument suggests that this process cannot generate a unique solution.

Thus, consulting with experts (professional translators) can be useful in generating richer qualitative evaluations of the three scales. To our knowledge, no previous studies have employed qualitative evaluation by professional translators. This could be because of a strong focus solely on quantitative psychometric measures, as only these are required to validate a questionnaire. However, some authors already recommended constituting expert committees when evaluating the equivalence between the original and translated versions of a scale (Guillemin et al., 1993; Tsang et al., 2017). Our study follows this rationale by asking professional translators specific questions aimed at comparing the equivalence between the original IRI and each of the three translations. This also aligns with previous studies in which the item wording of different translations of the same measure was briefly evaluated by researchers (Boussat et al., 2021; Perneger et al., 1999). In these studies, the authors focused especially on wording discrepancy, the use of more/less extreme terms with respect to the original version, which highlights a distancing from the original sense. Our study expands these previous evaluations by focusing on emotional intensity, wording form according to oral or written communication, and comparisons across translated versions. Additionally, the major novelty introduced by our study consists of interviews with independent translators.

### Materials and Methods

#### Participants

For the purposes of Study 2, six professional translators (four women and two men) were recruited from the co-authors’ network and former students who had obtained a degree in translation and interpreting studies. All translators were native French speakers. Four translators were born in France (two from the Ile-de-France region, two from the Hauts-de-France region), one in Belgium, and one in another European country with French-speaking parents. All attended professional training either in Belgium or France, and they had at least one year of translation experience. They were familiar with translating texts addressed to European French-speaking countries (France, Belgium, French-Swiss). They declared to translate texts in several domains (juridical, institutional, sport, medical, business, information science). The translators were remunerated for their participation in Study 2.

#### Procedure

Each translator received the original English version of the IRI and the three anonymized French versions. The translators were asked to evaluate the three French versions item by item.

First, for each item, translators were asked to order each of the three versions according to the degree to which it matched the original (“*Which version sounds better when compared to the original English version?*” and “*Which version sounds worst when compared to the original English version?”*). They could also annotate whether they considered that two or more versions sounded equally good/bad.

Second, translators were asked to declare, for each version, whether they considered the item appropriate for oral or written communication (“*Is this sentence something that you would say?*” and “*Is this sentence something that you would write?*”; yes/no answer), and whether they considered it equivalent to the original English version in terms of emotional intensity (“*Is the intensity of the sentence—the expressed action/emotion—equivalent to that of the original English version?”*; yes/no answer).

#### Data Analysis

The analyses were conducted to characterize inter-item differences in each French version of the IRI scale and among the three French versions. For each item within each French version (e.g., Gilet’s French translation of item #1), we computed the percentage of professional translators who considered that wording as unacceptable in the oral form, in the written form, and in terms of emotional intensity. Further, we computed the percentage of translators who considered the wording the worst among the three French versions (Braun et al., 2015; Gilet et al., 2013; Guttman & Laporte, 2000). We considered an item’s wording (e.g., Gilet’s translation of item #1) problematic when the majority of translators (>50%) considered it unacceptable in at least two parameters (oral form, written form, or intensity level) or when they considered it unacceptable in one parameter and evaluated it as the worst of the three French versions.

### Results

**Figure 1:**
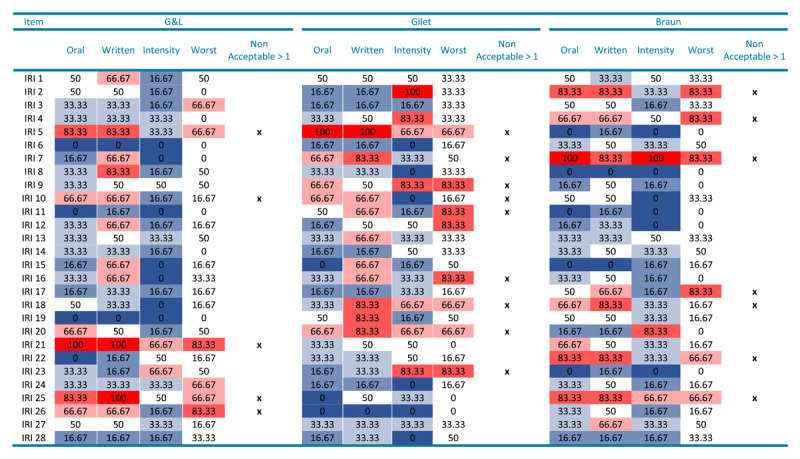
Item-by-Item Evaluation. Percentage of Translators (%) Considering as Non Acceptable each Item following the Established Criteria. *Note*. The color gradient represents the percentage of translators considering an item as being not acceptable with respect to the given criteria (% of “NO” answers). Darker red indicates a higher number of translators considering the item as being bad. Darker blue indicates a higher number of translators considering the item as being good. Oral = oral form appropriateness (“*Is this sentence something that you would say?*”; Written = written form appropriateness (“*Is this sentence something that you would write?*”; Intensity = emotional intensity appropriateness (“*Is the intensity of the sentence – the expressed action/emotion – equivalent to that of the original English version?”*). Worst = % of translators judging the item as the worst when compared to the other French versions of the same item. *Non Acceptable* > 1 = Items that are considered on average as non acceptable at more than one criterion. G&L = IRI version by Guttman & Laporte, 2000; Gilet = IRI version by Gilet et al., 2013; Braun = IRI version by Braun et al., 2015.

Figure 1 illustrates the results of the item-by-item evaluations on the basis of different criteria, namely the appropriateness of item wording in the oral form, written form, and emotional intensity. For each established criterion and for each item, we calculated the percentage of translators who considered the items as unacceptable (percentage of “no” answers to the corresponding question). The red color indicates that the majority of translators (>50%) evaluated the items as problematic in a specific parameter. This method resulted in Gilet’s version being rated as slightly superior with respect to the oral form relative to the other two versions (the number of problematic items in the “Oral” column was five, against six of Guttman and Laporte’s items, and seven of Braun’s). However, Gilet’s version had far more problematic items (seven) regarding intensity equivalence compared with Braun’s (three) and Guttman & Laporte’s (two). All versions were considered less appropriate in their written than oral form, with ten items evaluated as problematic in both Gilet’s and Guttman & Laporte’s versions and eight problematic items in Braun’s. Overall, nine items were considered problematic in more than one criterion (oral form, written form, emotional intensity) in Gilet’s version, seven in Braun’s version, and five in Guttman & Laporte’s version, with some overlaps across versions.

Table 7 highlights the most problematic items, relying on data from both Studies 1 and 2. Specifically, it summarizes the items’ kurtosis and skewness (Table 2), factorial loadings (Table 5), and translators’ evaluations (Figure 1). Table 7 confirms the lower psychometric and qualitative properties of Gilet’s version (highest number of items with high skewness or kurtosis; highest number of items judged as problematic by translators), resulting in a total of four items considered problematic for more than one parameter. Braun’s and Guttman & Laporte’s versions were more balanced in their strengths and weaknesses, with both having only one item deemed problematic for more than one parameter.

**Table 7:** Problematic Items when Considering Parameters assessed in Study 1 and 2.


ITEM	G&L				GILET				BRAUN			
		
	SKEW	KRT	CFA	TRANSL	SKEW	KRT	CFA	TRANSL	SKEW	KRT	CFA	TRANSL

IRI 1								X				X

IRI 2												X

IRI 3												

IRI 4			X									X

IRI 5				X				X				

IRI 6												

IRI 7									X			X

IRI 8							X			X		

IRI 9							X			X		

IRI 10				X				X				

IRI 11									X		X	

IRI 12		X				X	X			X		

IRI 13			X									

IRI 14						X	X					

IRI 15												

IRI 16		X				X		X				

IRI 17		X										X

IRI 18	X				X	X	X	X	X	X		X

IRI 19												

IRI 20								X				

IRI 21				X								

IRI 22												X

IRI 23								X				

IRI 24												

IRI 25		X		X								X

IRI 26				X								

IRI 27												

IRI 28												

Sum	2	3	2	5	3	5	2	9	2	4	1	7

Total	12				19				14			

Items problematic at >1 parameter:												

	Item #25				Items #12, #14, #16, #18				Item #18			


*Note*. G&L = IRI version by Guttman & Laporte, 2000; Gilet = IRI version by Gilet et al., 2013; Braun = IRI version by Braun et al., 2015. Skew = skeweness; Krt = kurtosis; CFA = Confirmatory Factor Analysis (involving Davis’ 4-factor model). Transl = Translation evaluation provided by the 6 translators following the established criteria (see Figure 1). An item was considered as problematic when meeting at least one of the following conditions: a) skewness or kurtosis value > |1|; b) loadings yielded by the CFA ≤ .40; c) resulting problematic at 2 or more translation criteria in Study 2.

## Discussion

The present research compared the three French versions of the Interpersonal Reactivity Index (IRI), one of the most used self-report measures of empathy. These three different translations of the IRI were independently provided by researchers from three different French-speaking regions: Canada (Guttman & Laporte, 2000), Switzerland (Gilet et al., 2013), and Belgium (Braun et al., 2015). No study had previously tested these three French versions with the same population or evaluated the similarities and differences in their item wording. To this aim, we conducted two studies: a repeated-measure study on a sample of French speakers living in France (Study 1) and an evaluation study of item wording with six professional translators (Study 2). Our research provides practical guidelines for researchers administering the IRI questionnaire in its French language form.

Results from the repeated-measure study (Study 1) revealed that the three versions, despite their differences in item wording, share good and almost identical psychometric properties. Indeed, statistical analyses demonstrated similar mean scores across the three versions, negligible floor and ceiling effects, good internal consistency of the subscales in each version, and acceptable and almost identical fit-indexes for the 4-factor model of the original American English version (Davis, 1983). The similar correlations found between the IRI scores and the other psychological variables of interest (moderate correlation between self-reported depression, anxiety, and PD; moderate to strong correlation between the Empathy Quotient and EC and PT) also supported similar discriminant and convergent validity.

These results corroborate previous studies exploring the associations between these measures. For instance, a previous study with the French population (Berthoz et al., 2008) found that IRI PD was the only IRI component that did not correlate with EQ total score, suggesting that IRI and EQ are two different measures of empathy, the first including personal distress in its conceptualization and the second excluding it. Specifically, it is important to highlight that IRI PD assesses personal distress in negative emotional situations (“*I sometimes feel helpless when I am in the middle of a very emotional situation*”) or in emergency situations, often involving another person (e.g., “*In emergency situations, I feel apprehensive and ill-at-ease*”). The moderate correlation of IRI PD with trait anxiety (between 0.46 and 0.52) is therefore understandable, although these two measures could not be said to assess exactly the same construct, given that trait anxiety represents a more generic state.

As already suggested by Murphy et al. (2020), future studies should examine the construct validity of PD as an index of empathy. Our results on the association between anxiety and empathy are in line with a recent meta-analysis (Nair et al., 2024) revealing that anxiety is often associated with greater fantasy and personal distress. In our sample, IRI PD was also the only IRI subscale to be positively correlated with depression (BDI), aligning with the results of a previous meta-analysis on the relationship between empathy and depression (Yan et al., 2021). Whether to include personal distress and fantasy in models of empathy or not is still a matter of debate. Recent evidence (Gaggero et al., 2023; Grynberg & López-Pérez, 2018) seems to support Batson et al.’s theoretical model (1987), which rejects the idea that higher negative arousal (personal distress) is a precursor of prosocial behavior.

Besides this theoretical debate, it is worth noticing that, in our sample, the three French adaptations of the IRI presented similar correlations with external variables, and these results concur with previous meta-analytical literature (Nair et al., 2024; Yan et al., 2021). Further, the correlation with external variables found in our study leads us to the same conclusion as Nair et al. (2024), namely that the practice of combining FS and PT to constitute a unique cognitive factor and EC and PD to constitute a unique affective factor is not ideal, “as it may obscure nuances in the components of empathy most relevant to psychopathology” (Nair et al., 2024).

Based on these results, the three French versions can be considered satisfactory for assessing empathy multi-dimensionally. This finding is quite reassuring since it suggests that the results obtained with different versions of the French IRI are comparable. Nonetheless, authors should be aware of the differences in the item wording of each version. Indeed, previous studies that compared different adaptations of the same instrument—the International Quality of Life Assessment questionnaire—suggested that respective imperfections in item wording in each translation may cancel each other out so that, by chance, the final psychometric properties appear to be similar (Perneger et al., 1999).

At this stage, one may wonder whether the three versions are equal and, therefore, interchangeable. To provide a definitive answer, it is essential to combine a quantitative and qualitative item-level analysis of each version with a more general analysis to better grasp the differences among the three, as in Perneger et al. (1999). Thus, in Study 2, we asked six professional translators to evaluate the wording of the items in each version with respect to several parameters. This elicited interesting information about substantial variations between the three versions. For instance, Gilet’s version was judged as being acceptable only in its oral form. However, it was also considered the least similar to the original American English version in terms of emotional intensity and was the version most frequently evaluated as the worst of the three (42%). In contrast, fewer differences were found between Braun’s and Guttman & Laporte’s versions. Specifically, the results demonstrated a slight preference for Braun’s version in its written form and a slight preference for Guttman & Laporte’s version with respect to its emotional intensity equivalence with the original American English version. When combining the item-level qualitative and quantitative information of Studies 1 and 2, it appears that the Gilet version presented a higher number (four) of problematic items based on cumulative criteria (items’ kurtosis, loadings, translators’ evaluations). On the contrary, no clear preference for Braun’s or Guttman & Laporte’s versions emerged since both versions presented different but balanced strengths and weaknesses. Guttman & Laporte’s (2000) appeared to be the preferred version based on translators’ evaluations, although that by Braun et al. (2015) exhibited slightly better factor loadings relying on the 4-factor model proposed by Davis. Consequently, we suggest using either Guttman & Laporte’s or Braun’s version. It is worth mentioning that these two original versions do not use the same labels in the Likert scale (as opposed to our identical labels across the three versions), suggesting the necessity to further compare these two questionnaires with their initial scales.

If researchers decide to use the Guttman & Laporte version, we suggest to modify items #13 and #25 to improve this version. In Guttman & Laporte version, the factor loading of item #13 (*“When I see someone get hurt, I tend to remain calm”*) was extremely low (.22) when compared with that of the other two versions. Therefore, we suggest that researchers using Guttman & Laporte’s version should change the formulation of item #13 to that proposed by Braun et al. (2015) (*“Quand je vois quelqu’un se blesser, j’ai tendance à garder mon calme.”*). Regarding item #25 (*“When I’m upset at someone, I usually try to put myself in his shoes for a while”*), the formulation used in Guttman & Laporte’s version showed both high kurtosis and received a bad overall evaluation from translators. It should, therefore, be substituted with the one used by Gilet et al. (2013) (*“Quand je suis en colère contre quelqu’un, j’essaie de me mettre à sa place pendant un moment”*), which the translators evaluated as the best (see Figure 1). These modifications applied to items #13 and #25 might further enhance the qualitative and psychometric properties of Guttman & Laporte’s adaptation.

Researchers willing to use Braun’s version could consider amending the formulation of item #18 (*“When I see someone being treated unfairly, I sometimes don’t feel very much pity for them”*) and replacing it with the formulation used by Guttman & Laporte (2000) (“*Il m’arrive de ne pas éprouver de pitié pour des personnes que je vois être traitées injustement*”), which had the best results across different parameters.

The translators’ feedback and comments revealed that Braun’s translations were usually judged as more pleasant to read, more linguistically correct, and used less familiar and more sophisticated vocabulary. Guttman & Laporte’s translations were usually more literal (e.g., item #21), but most of the time, they were also closest to the English formulation in terms of emotional intensity. These considerations can be useful when a French adaptation of the IRI is administered to a sample with a low educational level or with a limited vocabulary. Therefore, Guttman & Laporte’s version might be the easiest for participants with a medium-low level of education to complete or could be the best choice when comparing two groups with different educational levels, to reduce the risk of bias.

Irrespective of the version of the French adaptation used, it is important to highlight that the IRI, as a self-report instrument, could be prone to social desirability and self-deceptive biases (Saroglou et al., 2005). Social desirability measures were not included in this study due to survey length limitations. However, extremely high skewness values in item distribution (Table 2) can be a sign that an item is formulated in a way that elicits socially acceptable answers (e.g., item #18: *“When I see someone being treated unfairly, I sometimes don’t feel very much pity for them”*). Overall, we suggest using the IRI in combination with other self-report measures of empathy (e.g., the Empathic Quotient) or experimental tasks (MET, Dziobek et al., 2008) to minimize the effects of the desire to present a favorable public image of oneself or the unconscious tendency to provide honest but positively biased responses (Braun et al., 2015; Gaggero et al., 2024).

## Limitations

Some limitations of this study must be mentioned. First, the sample was not balanced in terms of gender. This could have partially affected the results of measurement invariance across genders, potentially causing an overestimation of the goodness of fit indexes based on the overrepresentation of the female group. Evidence of measurement invariance should be replicated in future studies with more balanced samples. Further, we adopted a repeated-measure design, justified by the good test-retest reliability of the IRI (*r* > 0.76) based on a recent meta-analysis (Lima & Osório, 2021). However, given Study 1’s repeated-measure design (two-month intervals between the three phases), the common phenomenon of sample attrition was observed. Future studies using a similar design should consider reducing the time interval between administration phases to limit the occurrence of this phenomenon. Future research should also consider evaluating the validity of the three French IRI versions in clinical populations, which might help researchers and clinicians select the most appropriate version. Regarding Study 2, future studies might consider increasing the number of interviewed professional translators to increase the reliability of their evaluations. Further, the translators in this study were all active in European French-speaking countries, mostly in France. Future studies could investigate whether their judgments can be replicated with a larger sample of translators coming from and working on documents for a variety of French-speaking countries. Finally, we acknowledge that the three French translations of the IRI differ not only in terms of item wording but also in the type of Likert scale used (see Table S1 of the Supplementary Material). In the present research we kept the same Likert scale across versions. Although Gilet et al. (2013) used a finer-grained 7-point Likert scale, we argue that adopting the 5-point Likert scale is most appropriate for recreating the same conditions of the original American English version of the questionnaire and is consistent with the other linguistic adaptations of the IRI. A 7-point Likert scale can enhance specificity but can also increase the burden on the respondents. A 5-point Likert scale is recommended for unipolar items (Boateng et al., 2018), and it is easier to visualize, which can be beneficial when testing specific populations (e.g., young adolescents or people with low educational levels). Despite some limitations, the present study has merit in examining three different versions of the IRI within the same sample with a repeated-measure design. Additionally, the combination of qualitative and quantitative methods helped us to illustrate the strengths and weaknesses of each version and to provide evidence-based advice to researchers wanting to use a French adaptation of the Interpersonal Reactivity Index.

## Conclusion

In this study, we compared three French adaptations of the Interpersonal Reactivity Index (IRI), namely Braun et al.’s (2015), Gilet et al.’s (2013), and Guttman & Laporte’s (2000) versions. Results from this first study conducted on a French sample demonstrated acceptable psychometric properties for all three versions. However, a finer-grained item-level analysis, in conjunction with evaluations provided by six independent translators, led to a slight preference for the item wording of Braun et al.’s (2015) and Guttman & Laporte’s (2000) versions (see Table S8 of the Supplementary Material). Whether to choose one or the other will also depend on an evaluation of participants’ educational levels since the two versions differ in vocabulary sophistication. Further, we provided recommendations for solving the issue of some problematic items in order to improve the overall quality of both versions.

## Data Accessibility statement

Data and data analysis scripts are available online (https://osf.io/k92rx/).

## Additional File

The additional file for this article can be found as follows:

10.5334/pb.1328.s1 Supplementary Material. Supplementary Tables S1–S8.
