# Supplementary material for: Assessing the French Interpersonal Reactivity Index (IRI): Psychometric and Qualitative Properties Through the Three French Versions of the IRI Scale
Source: Psychol Belg. 2025 Apr 2;65(1):69–86. doi: 10.5334/pb.1328 (PMC11967462; doi:10.5334/pb.1328)
Supplement: Supplementary Material. — Supplementary Tables S1–S8. [file pb-65-1-1328-s1.pdf]

Supplementary Material

Article Title:

Assessing the French Interpersonal Reactivity Index (IRI): Psychometric and Qualitative Properties of the Three French Versions of the IRI Scale

Supplementary Table 1 (S1)

Psychometric Properties of the Original English and the Three French Versions of the IRI Scale

		Original English	French Versions		
		Davis 1980;1983	G&L	Gilet	Braun
Questionnaire Characteristics	N. of Items	28 (7 per subscale)	28 (7 per subscale)	28 (7 per subscale)	28 (7 per subscale)<br>Short version: 15 items (3 for EC, 4 for the other subscales)
	Likert Scale Type & Coding	5-point scale<br>Coding: from 0 to 4<br>0 = “Does not describe me well”<br>4 = “Describes me very well”	5-point scale<br>Coding: from 1 to 5<br>1 = “Total disagreement”<br>5 = “Total agreement”	7-point scale<br>Coding: from 1 to 7<br>1 = “Does not describe me well”<br>7 = “Describes me very well”	5-point scale<br>Coding: from 0 to 4<br>0 = “Does not describe me well”<br>4 = “Describes me well”
Sample Characteristics	Nationality	U.S.	French -Speaking Canadian	French-Speaking Swiss	French-Speaking Belgian
	Population	Undergraduate University students	Diagnosed participants (AN, BDP), 1 CTL + family members	Non-clinical adults	University students
	Size	427	325	322	1244 + 729
Psychometric Properties	Cronbach’s alpha	EC = 0.72/.70<br>PD = 0.78/.78<br>PT = 0.75/78<br>FS = 0.78/.75	From .63 to .70	EC = 0.70<br>PD = 0.78<br>PT = 0.71<br>FS = 0.81	28-item version:<br>EC =.75, PD = .71, PT =.65, FS = .76<br>15-item version:<br>EC =.60, PD =.70, PT =.62, FS = .76
	Confirmatory Factor Analyis (4 factor)	-	-	CFI = .81, RMSEA = .065<br>SRMR = .07	28 item-version:<br>CFI = .81, RMSEA = 0.05<br>SRMR = 0.06<br>15-item version:<br>CFI = .92, RMSEA = 0.05,<br>SRMR = 0.05
	Divergent/Convergent validity	-	-	IRI x Social desirability (Crown-Marlow Scale). Results: CM x IRI EC *r* = .18***, CM x IRI PD *r* = -.24***, CM x IRI PT = ns, CM x IRI FS *r* = .32***	IRI x Empathy Quotient (EQ): EQ x IRI EC (r = .29**), EQ x IRI PD (r = -.26**), EQ x IRI PT (r = .35**), EQ x IRI FS = ns

Gender Differences	Females > Males in all subscales	Only females tested	Females > Males in EC, FS	Females > Males in EC, FS, PD

Note. G&L = IRI version by Guttman & Laporte, 2000; Gilet = IRI version by Gilet et al., 2013; Braun = IRI version by Braun et al., 2015. EC = Empathic Concern subscale; PD = Personal Distress subscales; PT= Perspective Taking subscale; FS= Fantasy subscale. CFA = Confirmatory Factor Analysis. CFI = Comparative Fit Index; RMSEA = Root Mean Square Error of Approximation; SRMR = Standardized Root Mean Residuals. BDP = Borderline Personality Disorder; AN =Anorexia Nervosa; CTL = Control Group.

Supplementary Table 2 (S2)

Internal Consistency (Cronbach's Alpha, McDonald's Omega) of the Three French Versions for the Clinical Subsample (N = 73)

Subscales	G&L	Gilet	Braun
PD	α = .82, ω = .91	α = .84, ω = .91	α = .81, ω = .89
EC	α = .81, ω = .87	α = .77, ω = .86	α = .85, ω = .90
FS	α = .82, ω = .89	α = .81, ω = .88	α = .84, ω = .92
PT	α = .78, ω = .84	α = .81, ω = .89	α = .79, ω = .86

Note. G&L = IRI version by Guttman & Laporte, 2000; Gilet = IRI version by Gilet et al., 2013; Braun = IRI version by Braun et al., 2015. EC = Empathic Concern subscale; PD = Personal Distress subscale; PT= Perspective Taking subscale; FS= Fantasy subscale.

Supplementary Table 3 (S3)

Descriptive and Inferential Statistics of IRI, STAI, BDI and EQ Scores as a Function of the Presence of a Clinical Diagnosis

Scale	Score	Diagnosis (n = 73)	No Diagnosis (n = 266)	*t-test*		
		M	M	t	p_{perm}	p_{adj}
G&L	Personal Distress (PD)	13.37	14.64	-1.93	.076	.380
	Empathic Concern (EC)	20.38	19.91	0.78	.402	.771
	Fantasy (FS)	19.01	19.52	-0.69	.498	.771
	Perspective Taking (PT)	18.79	18.24	0.87	.398	.771
Gilet	Personal Distress (PD)	12.33	13.02	-0.97	.328	.771
	Empathic Concern (EC)	20.66	20.41	0.42	.74	.854
	Fantasy (FS)	18.74	19.27	-0.74	.436	.771
	Perspective Taking (PT)	19.03	18.86	0.27	.812	.870
Braun	Personal Distress (PD)	13.15	13.60	-0.68	.514	.771
	Empathic Concern (EC)	19.66	19.88	-0.37	.722	.854
	Fantasy (FS)	19.12	19.38	-0.35	.716	.771
	Perspective Taking (PT)	19.04	19.02	0.43	.966	.966
**STAI - Trait**	Total Score	53.01	47.67	3.63	**.002****	**.015***
**BDI**	Total Score	9.75	6.79	3.65	**.002****	**.015***
EQ	Total Score	43.52	42.07	1.01	.292	.771

Note. G&L = IRI version by Guttman & Laporte, 2000; Gilet = IRI version by Gilet et al., 2013; Braun = IRI version by Braun et al., 2015. EC = Empathic Concern subscale; PD = Personal Distress subscale; PT= Perspective Taking subscale; FS= Fantasy subscale. STAI-T = Spielberger's Trait Anxiety Inventory; BDI = Beck Depression Inventory; EQ = Empathy Quotient; Diagnosis = group declaring having received a mental disorder

diagnosis; No Diagnosis = group declaring having received no mental disorder diagnosis. Non-parametric permutation t-tests between the groups were performed using the RVAideMemoire R package (Hervé and Hervé, 2020). Permutation tests are non-parametric tests that can be useful in case of deviation from normality and unequal sample sizes. p_{perm} = permutation p-value; p_{adj} = p-values adjusted using the Benjamini–Hochberg procedure. * = p <.05; ** = p <.01; *** = p <.001.

Supplementary Table 4 (S4) *Item-Total Correlations*

Item	G&L		Gilet		Braun	
	Item-Tot	Alpha.Without	Item-Tot	Alpha Without	Item-Tot	Alpha Without
1	0.30	0.80	0.27	0.81	0.27	0.83
2	0.47	0.79	0.48	0.80	0.42	0.82
3	0.25	0.80	0.23	0.81	0.28	0.83
4	0.21	0.80	0.33	0.81	0.42	0.82
5	0.42	0.79	0.47	0.80	0.45	0.82
6	0.26	0.80	0.33	0.81	0.32	0.83
7	0.40	0.79	0.33	0.81	0.37	0.82
8	0.19	0.80	0.21	0.81	0.17	0.83
9	0.22	0.80	0.31	0.81	0.27	0.83
10	0.35	0.79	0.29	0.81	0.41	0.82
11	0.41	0.79	0.37	0.80	0.45	0.82
12	0.32	0.80	0.32	0.81	0.29	0.83
13	0.23	0.80	0.22	0.81	0.26	0.83
14	0.45	0.79	0.39	0.80	0.50	0.82
15	0.15	0.80	0.25	0.81	0.22	0.83
16	0.42	0.79	0.42	0.80	0.43	0.82
17	0.25	0.80	0.32	0.81	0.30	0.83
18	0.37	0.79	0.22	0.81	0.28	0.83
19	0.16	0.80	0.20	0.81	0.25	0.83
20	0.52	0.79	0.45	0.80	0.50	0.82
21	0.24	0.80	0.24	0.81	0.22	0.83
22	0.47	0.79	0.44	0.80	0.52	0.82
23	0.53	0.79	0.48	0.80	0.51	0.82
24	0.25	0.80	0.30	0.81	0.32	0.83
25	0.20	0.80	0.26	0.81	0.27	0.83
26	0.48	0.79	0.52	0.80	0.54	0.82
27	0.21	0.80	0.23	0.81	0.24	0.83
28	0.25	0.80	0.34	0.81	0.34	0.83

Note. G&L = IRI version by Guttman & Laporte, 2000; Gilet = IRI version by Gilet et al., 2013; Braun = IRI version by Braun et al., 2015. Item-Tot = item-total scale correlation; Alpha.Without = scale's Cronbach's alpha without the item.

Supplementary Table 5 (S5)

Inter-item correlations in each version (Guttman & Laporte, 2000; Gilet et al. 2013; Braun et al., 2015)

a. Guttman & Laporte, 2000

	1	2	3	4	5	6	7	8	9	10	11	12	13	14	15	16	17	18	19	20	21	22	23	24	25	26	27
**1**																											
**2**	0.08																										
**3**	-0.06	0.18																									
**4**	-0.17	0.21*	0.25**																								
**5**	0.22**	0.15	0.03	0.03																							
**6**	0.05	0.05	-0.13	0.01	0.13																						
**7**	0.14	0.17	0.17	0.02	0.40***	0.12																					
**8**	0.03	0.24**	0.32***	0.02	0.05	-0.14	0.04																				
**9**	0.12	0.41***	0.04	0.12	0.08	-0.03	0.12	0.09																			
**10**	0.22**	0.08	-0.07	0.07	0.12	0.44***	0.13	-0.08	0.10																		
**11**	0.12	0.32***	0.44***	0.15	0.10	-0.14	0.12	0.47***	0.20	0.01																	
**12**	0.21*	0.12	0.06	0.03	0.57***	0.07	0.52***	-0.03	0.09	0.17	0.07																
**13**	0.03	0.20	0.08	0.15	0.07	0.10	0.14	-0.12	0.19	0.18	-0.00	0.06															
**14**	0.02	0.46***	0.27***	0.44***	0.14	-0.01	0.18	0.11	0.38***	0.15	0.27***	0.16	0.33***														
**15**	-0.03	0.12	0.23**	0.14	0.02	-0.16	0.05	0.41***	-0.01	-0.14	0.34***	-0.00	-0.07	0.13													
**16**	0.40***	0.10	0.00	-0.05	0.44***	0.16	0.35***	-0.03	0.04	0.17	0.08	0.40***	0.04	0.05	-0.00												
**17**	0.21*	0.01	-0.07	0.02	0.12	0.39***	0.06	-0.07	0.01	0.58***	0.00	0.07	0.14	0.01	-0.13	0.19											
**18**	0.01	0.48***	0.19	0.40***	0.05	-0.05	0.13	0.13	0.39***	0.09	0.24**	0.12	0.22*	0.58***	0.14	0.03	-0.03										
**19**	-0.04	-0.03	-0.08	-0.03	0.02	0.56***	-0.00	-0.16	-0.14	0.22*	-0.15	-0.01	0.08	-0.08	-0.08	0.05	0.24**	-0.09									
**20**	0.15	0.39***	0.21*	0.19	0.15	0.08	0.26***	0.17	0.28***	0.27***	0.31***	0.19	0.20	0.46***	0.14	0.23**	0.11	0.46***	-0.06								
**21**	0.12	0.18	0.34***	0.03	-0.04	-0.14	0.10	0.40***	0.10	-0.07	0.41***	-0.03	-0.04	0.13	0.31***	0.07	-0.12	0.11	-0.18	0.18							
**22**	0.13	0.30***	0.20*	0.22**	0.16	0.18	0.27***	0.06	0.12	0.29***	0.22*	0.19	0.14	0.36***	0.03	0.16	0.14	0.30***	0.08	0.45***	0.04						
**23**	0.32***	0.18	0.13	0.03	0.58***	0.08	0.51***	0.03	0.05	0.20*	0.18	0.57***	0.07	0.15	-0.01	0.61***	0.13	0.14	0.01	0.24**	0.09	0.29***					
**24**	0.12	0.02	-0.14	-0.05	0.14	0.59***	0.14	-0.21*	-0.13	0.32***	-0.15	0.06	0.12	-0.05	-0.19	0.23**	0.34***	-0.10	0.62***	0.03	-0.12	0.13	0.17				
**25**	0.04	0.23**	0.29***	0.09	-0.06	-0.19	-0.07	0.41***	0.08	-0.07	0.45***	-0.12	-0.10	0.09	0.32***	-0.00	-0.07	0.15	-0.11	0.13	0.41***	0.05	0.01	-0.16			
**26**	0.43***	0.13	0.11	-0.06	0.46***	0.08	0.36***	0.07	0.11	0.16	0.23**	0.32***	0.08	0.09	0.03	0.53***	0.17	0.06	0.02	0.23**	0.15	0.16	0.53***	0.17	0.15		
**27**	0.08	-0.01	-0.16	-0.10	0.06	0.54***	0.06	-0.12	-0.14	0.30***	-0.13	0.01	0.04	-0.09	-0.11	0.19	0.27***	-0.10	0.52***	0.03	-0.07	0.12	0.09	0.60***	-0.07	0.17	
**28**	0.03	0.26***	0.28***	0.19	-0.01	-0.14	0.00	0.34***	0.14	-0.05	0.43***	-0.09	-0.06	0.21*	0.23**	0.03	-0.08	0.26***	-0.08	0.19	0.38***	0.14	0.07	-0.09	0.45***	0.11	-0.11

Note: Pearson's correlation coefficients between the scale's items.

b. Gilet et al. 2013

	1	2	3	4	5	6	7	8	9	10	11	12	13	14	15	16	17	18	19	20	21	22	23	24	25	26	27
**1**																											
**2**	0.18																										
**3**	-0.07	0.20																									
**4**	-0.03	0.41***	0.27***																								
**5**	0.28***	0.19	0.09	0.08																							
**6**	0.05	0.02	-0.06	0.01	0.14																						
**7**	0.17	0.15	0.07	0.03	0.47***	0.05																					
**8**	0.10	0.14	0.28***	0.12	0.01	-0.10	-0.01																				
**9**	0.14	0.37***	0.13	0.22*	0.11	-0.07	0.07	0.18																			
**10**	0.15	0.05	-0.20	-0.07	0.14	0.40***	0.02	-0.02	0.17																		
**11**	0.04	0.26***	0.49***	0.19	0.11	-0.02	0.03	0.36***	0.23**	-0.05																	
**12**	0.14	0.12	0.08	0.04	0.50***	0.02	0.54***	0.01	0.14	0.06	0.06																
**13**	-0.03	-0.03	-0.05	0.11	0.04	0.42***	0.02	-0.15	0.02	0.19	-0.10	0.03															
**14**	-0.01	0.41***	0.26***	0.54***	0.14	-0.03	0.06	0.07	0.33***	0.02	0.23**	0.11	0.05														
**15**	0.02	0.26***	0.34***	0.27***	0.01	-0.09	0.05	0.39***	0.14	-0.13	0.22**	0.07	-0.06	0.23**													
**16**	0.23**	0.19	-0.04	0.08	0.43***	0.17	0.39***	-0.05	0.11	0.16	0.03	0.30***	0.16	0.04	0.06												
**17**	0.21*	0.04	-0.11	-0.10	0.12	0.40***	0.03	0.02	0.09	0.61***	0.05	0.00	0.20	-0.05	-0.08	0.21*											
**18**	0.03	0.32***	0.26***	0.39***	0.09	-0.07	0.16	0.21*	0.29***	0.05	0.18	0.17	-0.03	0.45***	0.20*	0.02	0.00										
**19**	-0.06	0.01	-0.10	0.01	0.02	0.53***	0.08	-0.18	-0.14	0.18	-0.08	0.03	0.48***	-0.05	-0.07	0.07	0.22**	-0.06									
**20**	0.15	0.27***	0.05	0.19	0.20	0.23**	0.16	0.10	0.18	0.33***	0.16	0.11	0.14	0.26***	0.09	0.24**	0.34***	0.23**	0.02								
**21**	0.11	0.21*	0.26***	0.08	0.10	-0.05	-0.04	0.44***	0.16	-0.03	0.38***	0.04	-0.08	0.10	0.24**	0.07	0.04	0.09	-0.18	0.15							
**22**	0.13	0.39***	0.28***	0.38***	0.16	0.13	0.13	0.21*	0.32***	0.14	0.29***	0.07	0.03	0.41***	0.19	0.05	0.12	0.35***	-0.02	0.39***	0.20						
**23**	0.21*	0.18	0.07	0.05	0.58***	0.13	0.54***	0.05	0.16	0.15	0.16	0.49***	0.04	0.13	0.02	0.61***	0.13	0.14	0.01	0.26***	0.11	0.22*					
**24**	0.05	-0.02	-0.15	-0.02	0.13	0.66***	0.10	-0.14	-0.08	0.36***	-0.13	0.01	0.53***	-0.10	-0.11	0.20	0.37***	-0.13	0.71***	0.16	-0.09	0.06	0.13				
**25**	0.10	0.32***	0.29***	0.21*	0.05	-0.09	-0.05	0.31***	0.07	-0.13	0.41***	-0.06	-0.11	0.16	0.35***	0.09	-0.06	0.13	-0.05	0.03	0.27***	0.16	0.02	-0.10			
**26**	0.33***	0.28***	0.13	0.08	0.47***	0.17	0.30***	0.08	0.17	0.17	0.25**	0.24**	0.06	0.09	0.12	0.48***	0.22**	0.11	0.05	0.25***	0.16	0.10	0.50***	0.12	0.18		
**27**	0.01	0.05	-0.17	0.01	0.08	0.43***	0.02	-0.20*	-0.08	0.30***	-0.09	-0.01	0.41***	-0.06	-0.11	0.23**	0.31***	-0.13	0.51***	0.11	-0.15	0.00	0.02	0.58***	-0.03	0.16	
**28**	0.08	0.32***	0.28***	0.22*	0.07	-0.04	0.01	0.41***	0.22*	-0.07	0.39***	-0.04	-0.12	0.22*	0.38***	0.03	0.01	0.26***	-0.08	0.12	0.34***	0.25**	0.04	-0.11	0.55***	0.23**	-0.06

Note: Pearson's correlation coefficients between the scale's items.

c. Braun et al. 2015

	1	2	3	4	5	6	7	8	9	10	11	12	13	14	15	16	17	18	19	20	21	22	23	24	25	26	27
1																											
2	0.11																										
3	-0.08	0.14																									
4	-0.00	0.36***	0.35***																								
5	0.25**	0.15	0.07	0.11																							
6	0.07	0.15	-0.12	0.05	0.13																						
7	0.13	0.08	0.11	0.19	0.40***	-0.01																					
8	0.04	0.03	0.31***	0.10	-0.05	-0.10	-0.03																				
9	0.07	0.23**	0.10	0.26***	-0.04	0.04	0.02	0.19																			
10	0.19	0.23**	-0.08	0.06	0.15	0.49***	0.11	-0.01	0.15																		
11	0.12	0.23**	0.46***	0.24**	0.11	0.01	0.13	0.48***	0.14	0.09																	
12	0.10	0.03	0.07	0.12	0.41***	0.05	0.46***	-0.09	0.02	0.10	0.11																
13	0.07	0.18	-0.04	0.07	0.09	0.25**	0.12	-0.13	-0.02	0.22*	-0.06	0.06															
14	0.01	0.39***	0.29***	0.62***	0.12	0.15	0.18	0.09	0.27***	0.18	0.21*	0.16	0.17														
15	-0.04	0.04	0.31***	0.25***	-0.01	-0.05	0.13	0.33***	0.08	-0.09	0.28***	0.10	0.03	0.21*													
16	0.33***	0.12	0.01	0.06	0.56***	0.08	0.34***	-0.07	0.04	0.15	0.09	0.25**	0.12	0.09	-0.02												
17	0.18	0.16	-0.04	-0.03	0.13	0.51***	0.05	-0.03	0.06	0.55***	0.06	0.04	0.17	0.04	-0.11	0.13											
18	0.06	0.24**	0.21*	0.44***	0.07	-0.02	0.27***	0.17	0.31***	0.05	0.24**	0.19	-0.05	0.41***	0.19	0.02	0.00										
19	0.05	0.10	-0.07	0.07	0.09	0.30***	0.06	-0.17	-0.06	0.23**	-0.08	0.10	0.51***	0.12	0.05	0.13	0.22*	-0.03									
20	0.10	0.43***	0.17	0.37***	0.16	0.23**	0.17	0.12	0.28***	0.33***	0.24**	0.13	0.17	0.50***	0.08	0.15	0.17	0.29***	0.07								
21	0.08	0.08	0.23**	0.06	0.07	-0.04	-0.02	0.33***	0.13	-0.05	0.38***	-0.01	-0.01	0.03	0.15	0.08	0.04	0.07	-0.06	0.01							
22	0.19	0.30***	0.21*	0.37***	0.19	0.15	0.19	0.15	0.24**	0.28***	0.30***	0.14	0.02	0.32***	0.26***	0.19	0.15	0.36***	0.07	0.34***	0.11						
23	0.27***	0.15	0.12	0.19	0.60***	0.13	0.42***	-0.03	0.06	0.18	0.18	0.38***	0.08	0.21*	0.04	0.65***	0.15	0.18	0.03	0.21*	0.17	0.29***					
24	0.14	0.17	-0.17	0.03	0.25***	0.39***	0.14	-0.22*	-0.10	0.35***	-0.02	0.10	0.46***	0.06	-0.15	0.22*	0.30***	-0.11	0.52***	0.16	-0.07	0.07	0.17				
25	0.05	0.12	0.33***	0.19	-0.00	-0.10	-0.04	0.43***	0.15	0.00	0.46***	0.00	-0.01	0.19	0.30***	-0.00	-0.05	0.16	-0.10	0.15	0.29***	0.22*	0.02	-0.10			
26	0.37***	0.18	0.11	0.13	0.49***	0.14	0.25**	0.05	0.09	0.19	0.25**	0.26***	0.12	0.14	0.07	0.61***	0.17	0.15	0.03	0.24**	0.20	0.25***	0.59***	0.21*	0.17		
27	0.12	0.11	-0.19	-0.07	0.12	0.36***	-0.05	-0.19	-0.11	0.33***	-0.04	-0.05	0.41***	-0.04	-0.15	0.22*	0.28***	-0.17	0.48***	0.10	-0.02	0.06	0.07	0.53***	-0.05	0.15	
28	-0.02	0.16	0.38***	0.25**	-0.00	0.01	0.06	0.38***	0.11	0.02	0.40***	0.03	-0.02	0.23**	0.29***	0.07	0.05	0.25**	-0.02	0.16	0.35***	0.23**	0.10	-0.09	0.48***	0.21*	-0.05

Note: Pearson’s correlation coefficients between the scale’s items.

Supplementary Table 6 (S6)

Measurement Invariance Results for Female (n = 261) vs Male (n = 71) Groups

Fit Indexes	χ2	*df*	CFI	TLI	RMSEA	SRMR	Δχ2	Δ*df*	*p*	ΔCFI	ΔMc
**G&L**											
Configural	1304.41***	688	0.804	0.785	0.073	0.084	-	-	-	-	-
Metric	1345.58***	712	0.079	0.786	0.073	0.087	41.17	24	.016 (*)	0.005	-0.006
Scalar	1378.61***	736	0.796	0.790	0.073	0.088	33.04	24	0.10	0.003	-0.017
**Gilet**											
Configural	1396.19***	688	0.780	0.759	0.079	0.085	-	-	-	-	-
Metric	1440.54***	712	0.774	0.760	0.079	0.089	44.35	24	0.006**	0.006	-0.006
Scalar	1489.64***	736	0.766	0.760	0.079	0.090	49.10	24	0.002**	0.008	0.001
**Braun**											
Configural	1274.43 ***	688	0.804	0.785	0.072	0.081	-	-	-	-	-
Metric	1310.91***	712	0.084	0.800	0.071	0.084	36.48	24	.049 (*)	0.004	-0.011
Scalar	1365.12***	736	0.790	0.790	0.072	0.085	54.21	24	< 0.001 (***)	0.010	0.140

Note. G&L = IRI version by Guttman & Laporte, 2000; Gilet = IRI version by Gilet et al., 2013; Braun = IRI version by Braun et al., 2015. χ2 = chi-squared statistics; *df* = degrees of freedom; CFI = Comparative Fit Index; TLI = Tucker-Lewis index, RMSEA = Root Mean Square Error of Approximation; SRMR = Standardized Root Mean Residuals; Mc = McDonald's noncentrality index.

Supplementary Table 7 (S7)

Descriptive statistics *of IRI, STAI, BDI and EQ Scores as a function of the Gender Declared*

Scale	Score	Females (n = 261)	Males (n = 71)	t-test		
		M	M	t	p_{perm}	p_{adj}
G&L	Personal Distress (PD)	14.49	13.82	1.00	.346	.346
	**Empathic Concern (EC)**	20.56	17.79	4.59	**.002****	**.007****
	Fantasy (FS)	19.75	18.34	1.88	.072	.108
	Perspective Taking (PT)	18.50	17.76	1.14	.262	.281
Gilet	Personal Distress (PD)	13.06	12.07	1.37	.184	.212
	**Empathic Concern (EC)**	21.01	18.30	4.76	**.002****	**.007****
	Fantasy (FS)	19.49	18.01	2.03	.036*	.077
	Perspective Taking (PT)	19.20	17.77	2.27	**.03***	.075
Braun	Personal Distress (PD)	13.82	12.31	2.29	**.03***	.075
	**Empathic Concern (EC)**	20.36	17.69	4.50	**.002****	**.007****
	Fantasy (FS)	19.64	18.20	1.95	.068	.108
	Perspective Taking (PT)	19.27	18.06	1.98	.044	.082
STAI	Total Score	49.27	46.79	1.64	.106	.136
BDI	Total Score	7.59	6.54	1.27	.210	.212
**EQ**	**Total Score**	44.25	35.53	6.42	**.002****	**.007****

Note. G&L = IRI version by Guttman & Laporte, 2000; Gilet = IRI version by Gilet et al., 2013; Braun = IRI version by Braun et al., 2015. STAI-T = Spielberger's Trait Anxiety Inventory; BDI = Beck Depression Inventory; EQ = Empathy Quotient. Non-parametric

permutation t-tests between groups (Females vs. Males) were performed using the RVAideMemoire R package (Hervé and Hervé, 2020). Permutation tests are non-parametric tests which are useful in case of deviation from normality and unequal sample sizes. Given the small proportion of participants selecting the option “other”/ “I prefer not to say” for gender (n = 7), we excluded these participants from the analysis of gender differences. p_{perm} = permutation p-value, p_{adj} = p-values adjusted using the Benjamini–Hochberg procedure. * = p <.05; ** = p <.01; *** = p <.001.

Supplementary Table 8 (S8) French Adaptations of the IRI by:

a. **Guttman & Laporte, 2000.**

N°	Items - Guttman & Laporte, 2000
1	Assez régulièrement, je rêvasse et fantasme à propos de choses qui pourraient m'arriver.
2	J'ai souvent des sentiments de tendresse, de compassion pour les personnes moins favorisées que moi.
3	Je trouve parfois difficile de voir les choses du point de vue de l'autre.
4	Il m'arrive de ne pas me sentir sincèrement désolé(e) pour les autres lorsqu'ils ont des problèmes.
5	Je deviens vraiment absorbé(e) par les sentiments des personnages d'un roman.
6	Dans les situations d'urgence, je me sens inquiet(e) et mal à l'aise.
7	Lorsque je regarde un film ou une pièce de théâtre, je suis généralement objectif(ve), et il est rare que je sois complètement pris(e) dedans.
8	En cas de désaccord, j'essaie de voir le point de vue de chacun avant de prendre une décision.
9	Lorsque je vois une personne se faire exploiter, j'éprouve un certain sentiment de protection envers elle/à son égard.
10	Je me sens parfois désarmé(e) lorsque je me trouve au cœur d'une situation très émotionnelle.
11	Parfois, j'essaie de mieux comprendre mes ami(e)s en imaginant comment les choses se présentent de leur point de vue.
12	C'est assez rare que je sois fortement absorbé(e) par un bon livre ou un bon film.
13	Quand je vois qu'on fait du mal à quelqu'un, j'ai tendance à garder mon calme.
14	D'habitude, les malheurs des autres ne m'affectent pas vraiment.
15	Si je suis sûr(e) d'avoir raison sur un point, je ne perds pas tellement de temps à écouter les arguments des autres.
16	Après avoir vu une pièce de théâtre ou un film, il m'est arrivé de me sentir comme si j'étais un des personnages.
17	Me trouver dans une situation de tension émotionnelle me fait peur.
18	Il m'arrive de ne pas éprouver de pitié pour des personnes que je vois être traitées injustement.
19	En général, je suis plutôt efficace dans les situations d'urgence.
20	Je suis souvent assez touché(e) par les événements que je vois se produire.
21	Je crois qu'il y a deux côtés à toute question et j'essaie de les regarder tous les deux.
22	J'aurais tendance à me décrire comme une personne au cœur tendre/sentimentale.

23	Lorsque je regarde un bon film, je peux très facilement me mettre à la place du personnage principal.
24	J'ai tendance à perdre le contrôle de moi-même dans les situations d'urgence.
25	Quand j'en veux à quelqu'un, j'essaie habituellement de me mettre 'dans sa peau' pendant un moment.
26	Lorsque je suis en train de lire une histoire intéressante, j'imagine ce que je ressentirais si les événements de l'histoire m'arrivaient.
27	Je perds mes moyens quand je vois quelqu'un qui a gravement besoin d'aide dans une situation d'urgence.
28	Avant de critiquer quelqu'un, j'essaie d'imaginer comment je me sentirais si j'étais à sa place.

b. Gilet et al., 2013

N°	Items – Gilet et al., 2013
1	Je rêve régulièrement tout éveillé(e) aux choses qui pourraient m'arriver.
2	J'éprouve souvent de la tendresse pour les gens moins chanceux que moi.
3	Je trouve parfois difficile de voir les choses du point de vue de quelqu'un d'autre.
4	Il m'arrive de ne pas être désolé(e) pour les gens qui ont des problèmes.
5	Je m'implique vraiment dans les sentiments ressentis par les personnages d'un roman.
6	Dans les situations d'urgence je suis inquiet(e) et mal a` l'aise.
7	Lorsque je regarde un film ou une pièce de théâtre, je suis généralement objectif(ve), et il est rare que je sois complètement pris(e) dedans.
8	Lors d'un désaccord, j'essaie d'écouter le point de vue de chacun avant de prendre une décision.
9	Quand je vois une personne dont on a profité, j'ai envie de la protéger.
10	Je me sens parfois désemparé(e) quand je me trouve au beau milieu d'une situation fortement émotionnelle.
11	J'essaie parfois de mieux comprendre mes amis en imaginant comment ils voient les choses de leur perspective.
12	Il est relativement rare que je me laisse prendre par un bon livre ou un bon film.
13	Quand je vois quelqu'un de blessé, j'ai tendance à rester calme.
14	D'habitude, je me soucie très peu du malheur des autres.
15	Si je suis sûr(e) d'avoir raison a` propos de quelque chose je ne perds pas mon temps a` écouter les arguments des uns et des autres.
16	Après avoir regardé un film ou une pièce de théâtre, c'est comme si j'étais l'un des personnages.
17	Être dans une situation de tension émotionnelle me fait peur.

18	Quand je vois quelqu'un être traité de façon injuste je ne ressens pas beaucoup de pitié pour lui.
19	D'habitude je suis plutôt efficace face aux situations d'urgence.
20	Je suis souvent touché(e), affecté(e) par les événements qui arrivent.
21	Je crois qu'il y a toujours deux facettes à chaque question ou problème et j'essaie de les prendre en compte toutes les deux.
22	Je me décrirais comme une personne au cœur tendre, plutôt compatissante.
23	Quand je regarde un bon film, je peux très facilement me mettre à la place du personnage principal.
24	J'ai tendance à perdre mes moyens dans des situations d'urgence.
25	Quand je suis en colère contre quelqu'un j'essaie de me mettre à sa place pendant un moment.
26	Quand je lis une histoire ou un roman intéressant, j'imagine ce que je ressentirais si les événements de l'histoire m'arrivaient.
27	En cas d'urgence, quand je vois quelqu'un qui a sérieusement besoin d'aide je m'effondre totalement.
28	Avant de critiquer quelqu'un j'essaie d'imaginer ce que je ressentirais si j'étais à sa place.

c. Braun et al., 2015

N°	Items - Braun et al., 2015
1	Je me surprends assez souvent à rêver et à fantasmer sur des choses qui pourraient m'arriver
2	Je suis souvent sensible et apitoyé(e) face à des gens moins chanceux que moi
3	J'ai parfois des difficultés à voir les choses du point de vue de l'autre
4	Parfois, je n'éprouve pas beaucoup de pitié quand d'autres gens ont des problèmes
5	Je me laisse complètement prendre par les sentiments des personnages d'un roman
6	Face à des situations critiques, je me sens inquiet(e) et mal à l'aise
7	Habituellement, je suis objectif (ve) quand je regarde un film ou une pièce et je suis rarement tout à fait emballé(e)
8	En cas de désaccord, j'essaie de tenir compte du point de vue de chacun avant de prendre une décision
9	Quand je vois quelqu'un se faire avoir, j'ai une certaine envie de le protéger
10	Quand je me retrouve dans une situation très émotionnelle, je me sens parfois désarmé(e)
11	J'essaie parfois de mieux comprendre mes amis en imaginant comment les choses se présentent de leur point de vue
12	Il est assez rare que je sois très pris(e) par un bon livre ou un bon film

13	Quand je vois quelqu’un se blesser, j’ai tendance à garder mon calme
14	D’habitude, les malheurs des autres ne me perturbent pas beaucoup
15	Si je suis certain(e) d’avoir raison à propos de quelque chose, je ne perds pas mon temps à écouter les arguments des autres
16	Après avoir vu une pièce de théâtre ou un film, il m’est arrivé de me sentir dans la peau d’un des personnages
17	Être dans une situation émotionnelle tendue m'effraie
18	Quand je vois quelqu’un se faire traiter injustement, il m’arrive de ne pas éprouver beaucoup de pitié pour lui
19	Je suis habituellement assez efficace pour gérer des situations d’urgences
20	Je suis souvent très touché(e) par des événements dont je suis témoin
21	Je crois qu’il y a deux facettes à chaque question et j’essaie de les considérer toutes les deux
22	Je pourrais me décrire comme quelqu’un au coeur assez tendre
23	Quand je regarde un bon film, je peux très facilement me mettre à la place d’un des personnages principaux
24	J’ai tendance à perdre mon sang froid dans des situations critiques
25	Quand je suis fâché(e) sur quelqu’un, j’essaie habituellement de me mettre à sa place pendant un moment
26	Quand je lis un roman ou une histoire intéressant(e), j’imagine comment je me sentirais si les événements de l’histoire m’arrivaient
27	Quand je vois quelqu’un qui a vraiment besoin d’aide dans une situation critique, je perds les pédales
28	Avant de critiquer quelqu’un, j’essaie d’imaginer comment je me sentirais si j’étais à sa place
